# Supplementary material for: Predicted structure of the complete CPLANE complex reveals novel interactors and mechanisms of Jbts17 during ciliogenesis
Source: bioRxiv. 2026 Oct 6:2026.10.05.756796. Preprint. [Version 1] doi: 10.64898/2026.10.05.756796 (PMC13692098; doi:10.64898/2026.10.05.756796)
Supplement: Supplementary file 1 [file NIHPP2026.10.05.756796v1-supplement-2.pdf]

bioRxiv preprint doi: https://doi.org/10.64898/2026.10.05.756796; this version posted October 6, 2026. The copyright holder for this preprint (which was not certified by peer review) is the author/funder, who has granted bioRxiv a license to display the preprint in perpetuity. It is made available under a CC-BY 4.0 International license.

Supplemental Figure 1. AlphaFold predicts the structure of JBTS17 and reveals similarities to other WDR–α–helical proteins.
(A) AlphaFold2-predicted structure of full length JBTS17. Residues are color-coded by predicted Local Distance Difference Test (pLDDT) scores.
(B) PAE plot for the full length JBTS17 model.
(C) AlphaFold2-predicted structure of IDR-trimmed JBTS17 ($JBTS17^{1–1768}$). Residues are color-coded by predicted Local Distance Difference Test (pLDDT) scores.
(D) PAE plot for the IDR-trimmed JBTS17 model.
(E–J) Structural comparison of proteins containing WDR beta-propellers followed by alpha-helical regions: (E) human JBTS17, (F) human IFT140, (G) human clathrin heavy chain, (H) human COP1β, (I) yeast Sec31, and (J) human WDPCP. The experimentally determined structure is shown for IFT140 (PDB 8BBF) and WDPCP from the solved partial CPLANE structure (PDB 7Q3D), while AlphaFold-predicted structures are shown for the remaining proteins.
(K) Structural superposition of the N-terminal WDR and alpha-helical regions of AlphaFold-predicted JBTS17 and experimentally determined human WDPCP (PDB 7Q3D), illustrating their similar domain architecture. Structures were superimposed using the ChimeraX Matchmaker tool.

bioRxiv preprint doi: https://doi.org/10.64898/2026.10.05.756796; this version posted October 6, 2026. The copyright holder for this preprint (which was not certified by peer review) is the author/funder, who has granted bioRxiv a license to display the preprint in perpetuity. It is made available under a CC-BY 4.0 International license.

Supplemental Figure 2. Structural comparison of the predicted CPLANE complex and mapping of JBTS17 variants of uncertain significance.
(A) Superposition of the AlphaFold2-predicted five-membered CPLANE complex with the experimentally determined partial human CPLANE structure (PDB 7Q3D). The experimental structure contains human WDPCP, INTU, and FUZ, but not JBTS17 or RSG1. Corresponding subunits are shown in matching colors, with the experimental structure shown in lighter colors. The structures were superimposed using the ChimeraX Matchmaker tool.
(B) Mapping of ClinVar missense variants of uncertain significance (VUS) onto the AlphaFold2-predicted CPLANE complex. JBTS17 residues containing VUS and located within 4 Å of another CPLANE subunit are highlighted in magenta and shown as spheres. Nineteen interface-associated VUS were identified after excluding contacts involving low-confidence/disordered regions omitted from the structural visualization.
(C-E) Enlarged views of selected predicted JBTS17 interfaces containing ClinVar VUS. (C) VUS located at predicted interfaces with FUZ and RSG1. (D) VUS located at the predicted interface with INTU. (E) VUS located at the predicted interface with WDPCP. VUS-containing JBTS17 residues are shown in magenta.

bioRxiv preprint doi: https://doi.org/10.64898/2026.10.05.756796; this version posted October 6, 2026. The copyright holder for this preprint (which was not certified by peer review) is the author/funder, who has granted bioRxiv a license to display the preprint in perpetuity. It is made available under a CC-BY 4.0 International license.

Supplemental Figure 3. Localization of additional predicted JBTS17 interactors in multiciliated cells.

(A-L) Representative images of *Xenopus* multiciliated cells expressing GFP-tagged candidate interactors. Cell membranes and basal bodies are marked by membrane-BFP and Centrin2-RFP, respectively. Scale bars = 10 μm.

bioRxiv preprint doi: https://doi.org/10.64898/2026.10.05.756796; this version posted October 6, 2026. The copyright holder for this preprint (which was not certified by peer review) is the author/funder, who has granted bioRxiv a license to display the preprint in perpetuity. It is made available under a CC-BY 4.0 International license.

Supplemental Figure 4. Predicted structures of candidate JBTS17 interactions identified by the AlphaFold screen.

AlphaFold2-Multimer-predicted structures of candidate interactions identified in the JBTS17 protein-protein interaction screen and discussed in the text. Each model contains the JBTS17 fragment used in the screen together with the indicated candidate interactor. JBTS17 fragments are shown in gold and candidate proteins are shown in green.

(A) $JBTS17^{CIDR}$-CSNK1A1
(B) $JBTS17^{BLD}$-ROPN1L
(C) $JBTS17^{WDR}$-EZR
(D) $JBTS17^{CIDR}$-AHI1
(E) $JBTS17^{CIDR}$-PLK1
(F) $JBTS17^{CIDR}$-POC1A
(G) $JBTS17^{CIDR}$-POC1B
(H) $JBTS17^{Helix1}$-BBS4
(I) $JBTS17^{WDR}$-NME5
(J) $JBTS17^{Helix2}$-MDH1B
(K) $JBTS17^{BLD}$-ACTR1A
(L) $JBTS17^{WDR}$-ATG16L1
(M) $JBTS17^{WDR}$-SQSTM1
(N) $JBTS17^{Helix2}$-RAB8A
(O) $JBTS17^{Helix2}$-RAB10

bioRxiv preprint doi: https://doi.org/10.64898/2026.10.05.756796; this version posted October 6, 2026. The copyright holder for this preprint (which was not certified by peer review) is the author/funder, who has granted bioRxiv a license to display the preprint in perpetuity. It is made available under a CC-BY 4.0 International license.

Supplemental Table 1. Predicted JBTS17 protein interactions and target protein annotations.
Results of the structure-based interaction screen between five JBTS17 fragments and candidate ciliary proteins. The “All Results” sheet contains the combined screen results, while the “WDR,” “Helix1,” “Helix2,” “BLD,” and “C-IDR” sheets contain results for each individual JBTS17 bait fragment. Each row represents a predicted bait–target interaction and includes the target protein name, primary gene name, interaction confidence scores (ipSAE, ipTM, ipSAE_d0chn, ipSAE_d0dom, ipTM_d0chn, pDockQ, and pDockQ2), and target annotations. The “RFX2 Target,” “Toriyama APMS Hit,” and “CiliaCarta Member” columns indicate whether the target belongs to each respective candidate protein set. The “Annotations” sheet provides additional target information, including protein identifiers, ortholog annotations, protein lengths, predicted disordered regions, and Gene Ontology biological process annotations.

bioRxiv preprint doi: https://doi.org/10.64898/2026.10.05.756796; this version posted October 6, 2026. The copyright holder for this preprint (which was not certified by peer review) is the author/funder, who has granted bioRxiv a license to display the preprint in perpetuity. It is made available under a CC-BY 4.0 International license.

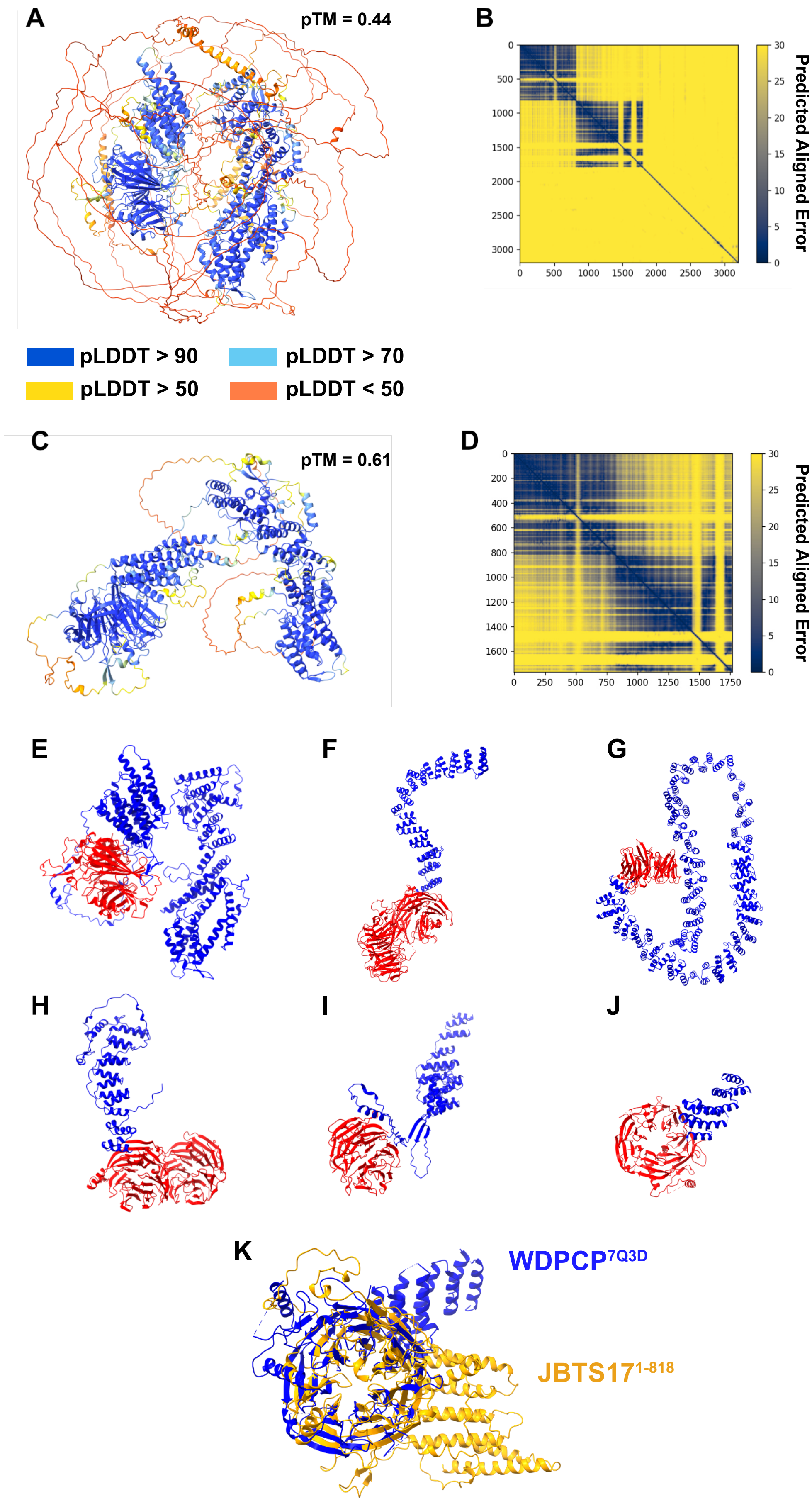

Gardner *et al.*,
Supp. Figure 1

bioRxiv preprint doi: https://doi.org/10.64898/2026.10.05.756796; this version posted October 6, 2026. The copyright holder for this preprint (which was not certified by peer review) is the author/funder, who has granted bioRxiv a license to display the preprint in perpetuity. It is made available under a CC-BY 4.0 International license.

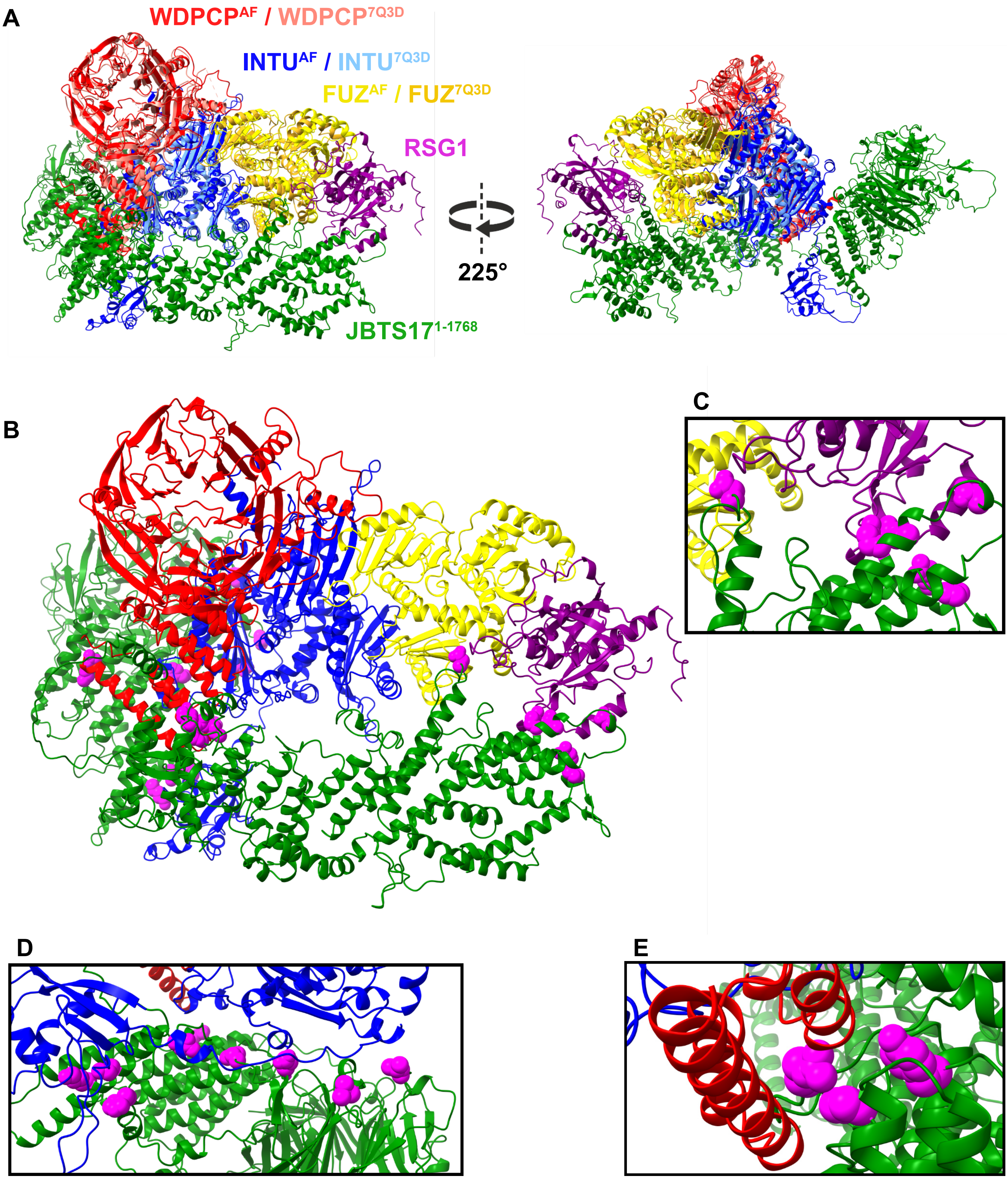

Gardner *et al.*,
Supp. Figure 2

bioRxiv preprint doi: https://doi.org/10.64898/2026.10.05.756796; this version posted October 6, 2026. The copyright holder for this preprint (which was not certified by peer review) is the author/funder, who has granted bioRxiv a license to display the preprint in perpetuity. It is made available under a CC-BY 4.0 International license.

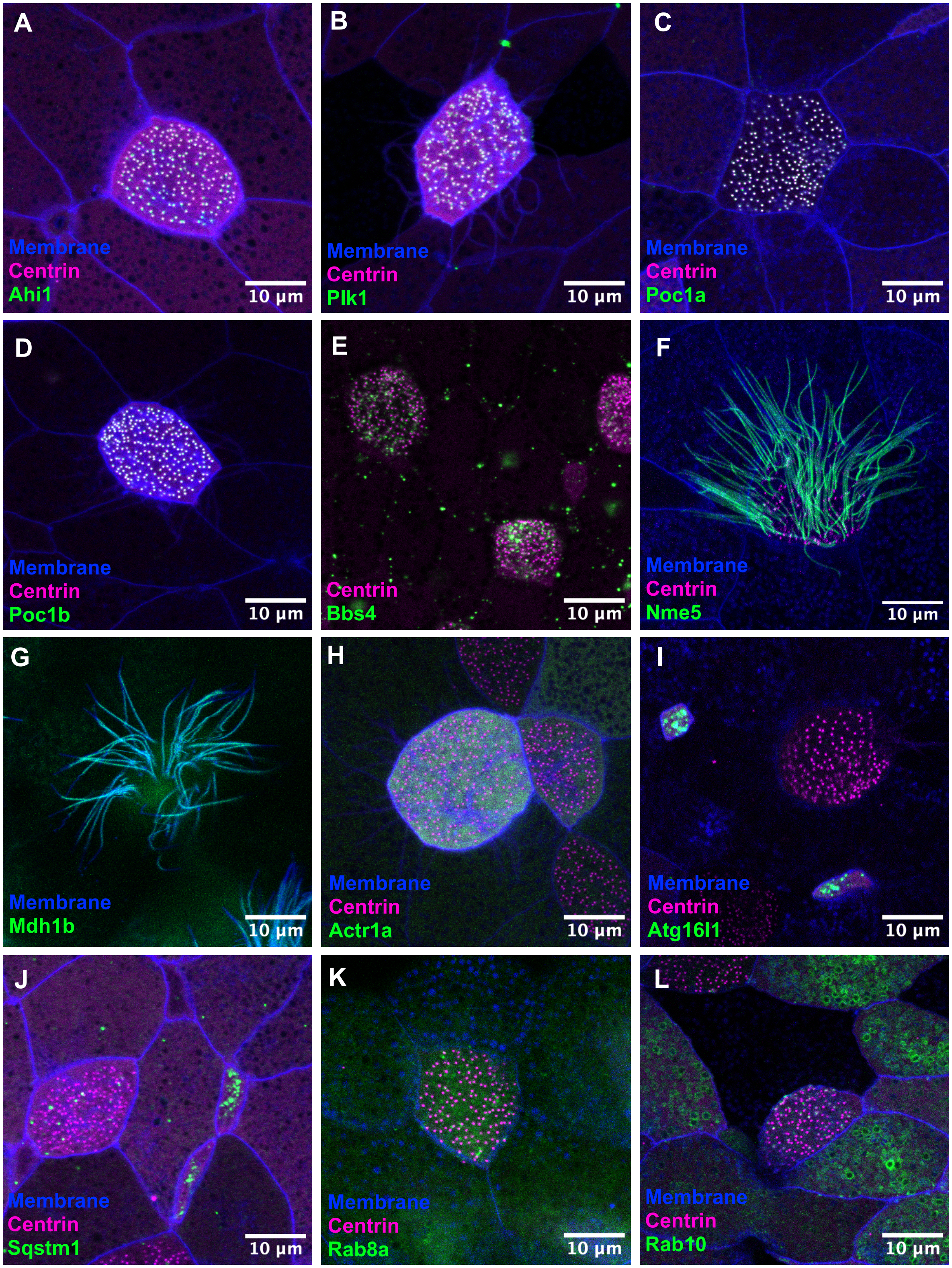

Gardner *et al.*,
Supp. Figure 3

bioRxiv preprint doi: https://doi.org/10.64898/2026.10.05.756796; this version posted October 6, 2026. The copyright holder for this preprint (which was not certified by peer review) is the author/funder, who has granted bioRxiv a license to display the preprint in perpetuity. It is made available under a CC-BY 4.0 International license.

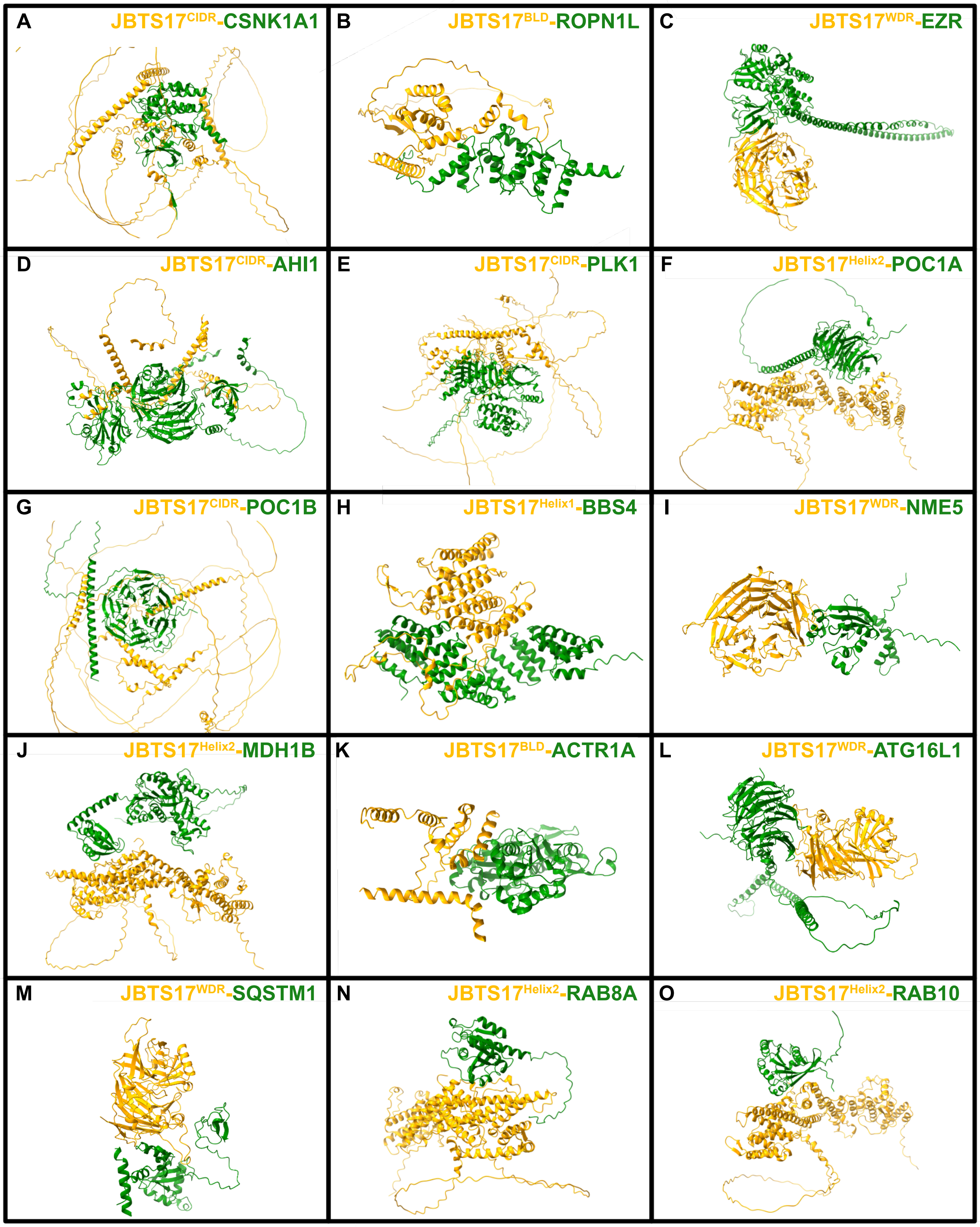

Gardner *et al.*,
Supp. Figure 4
